# Effects of zinc transporters on *Cryptococcus gattii* virulence

**DOI:** 10.1038/srep10104

**Published:** 2015-05-07

**Authors:** Rafael de Oliveira Schneider, Camila Diehl, Francine Melise dos Santos, Alícia Corbellini Piffer, Ane Wichine Acosta Garcia, Marcos Iuri Roos Kulmann, Augusto Schrank, Lívia Kmetzsch, Marilene Henning Vainstein, Charley C. Staats

**Affiliations:** 1 Centro de Biotecnologia; 2 Departamento de Biologia Molecular e Biotecnologia, Universidade Federal do Rio Grande do Sul, Av. Bento Gonçalves 9500, 43421, Caixa Postal 15005, Porto Alegre, RS 91501-970, Brazil

## Abstract

Zinc is an essential nutrient for all living organisms because it is a co-factor of several important proteins. Furthermore, zinc may play an essential role in the infectiousness of microorganisms. Previously, we determined that functional zinc metabolism is associated with *Cryptococcus gattii* virulence. Here, we characterized the ZIP zinc transporters in this human pathogen. Transcriptional profiling revealed that zinc levels regulated the expression of the *ZIP1*, *ZIP2* and *ZIP3* genes, although only the *C. gattii* zinc transporter Zip1 was required for yeast growth under zinc-limiting conditions. To associate zinc uptake defects with virulence, the most studied cryptococcal virulence factors (i.e., capsule, melanin and growth at 37 °C) were assessed in *ZIP* mutant strains; however, no differences were detected in these classical virulence-associated traits among the mutant and WT strains. Interestingly, higher levels of reactive oxygen species were detected in the *zip1*Δ and in the *zip1*Δ *zip2*Δ double mutants. In line with these phenotypic alterations, the *zip1*Δ *zip2*Δ double mutant displayed attenuated virulence in a murine model of cryptococcosis. Together, these results indicate that adequate zinc uptake is necessary for cryptococcal fitness and virulence.

The basidiomycete yeasts *Cryptococcus neoformans* and *Cryptococcus gattii* are the etiological agents of cryptococcosis, a life-threatening disease that is generally characterized by meningoencephalitis. Cryptococcal infections are responsible for approximately 1 million cases of meningoencephalitis annually, resulting in approximately 625,000 deaths, principally in HIV-infected individuals^1^. Although *C. neoformans* infects mainly immunocompromised patients, *C. gattii* can cause disease in immunocompetent patients^2^,^3^. Recently, *C. gattii* has gained attention due to an outbreak that occurred in Canada and spread into the United States^4^.

A wide range of strategies is used by the immune system to control the proliferation of infectious agents. For example, macrophages and other phagocytes produce a harsh intracellular environment that is unfavorable for microbial development. This cellular compartment is characterized by extremely low pH and the presence of reactive nitrogen and oxygen species (ROS), enzymes and antimicrobial peptides^5^. Additionally, the host immune system can restrict the availability of essential metals in a process called nutritional immunity. One example is the restriction of iron availability, which is a well-characterized defense mechanism against infections^6^. However, nutritional immunity is not limited to iron withholding^7^. Zinc is an essential element for all organisms and is required for the functions of many proteins with catalytic and structural roles^8^. Recently, we demonstrated that the proper regulation of zinc metabolism is important for the virulence of the human pathogen *C. gattii* because the absence of the master zinc metabolism regulator Zap1 impaired the virulence of this pathogen^9^. Furthermore, an association between the regulation of zinc metabolism and virulence in pathogenic fungi was observed in *Aspergillus fumigatus* and *Candida albicans*, where the inactivation of Zap1 orthologs impaired the infection process^10^,^11^.

Fungal zinc homeostasis has been best characterized in the model organism *Saccharomyces cerevisiae*^12^,^13^, in which proteins of the ZIP family of zinc transporters take up this metal. ZIP zinc transporters are found in all life domains and were named based on the first identified members (Zrt1p from *S. cerevisiae* and IRT-like protein from *Arabidopsis thaliana*). While Zrt1p was characterized as a zinc transporter, IRT were able to transport iron^14^. Zinc metabolism is a potential target for the development of antifungal drugs because some fungal pathogens demonstrate greater sensitivity to deprivation of zinc than iron^15^. Moreover, recent work demonstrated that some drugs led to alterations in the expression of zinc homeostasis-related genes in *S. cerevisiae*^16^. Despite the importance of *C. gattii* as a pathogen, zinc metabolism is still poorly characterized in this fungus. Here, we describe the functional characterization of ZIP zinc transporters in *C. gattii* R265, their role in zinc metabolism and the effects of gene deletion on cryptococcal virulence.

## Results

### Identification of ZIP transporters in *C. gattii*

Analysis of the *C. gattii* predicted proteome^17^ for a PFAM domain ZIP zinc transporter (PF02535) revealed the presence of 4 four different genes. Previously, we showed that intracellular zinc levels altered the transcript levels of three genes (CNAG_6066 – *ZIP1*, CNBG_2209 – *ZIP2*, and CNBG_5361 – *ZIP3*)^9^. The fourth gene (CNBG_3633 (*ZIP4*)) is a direct ortholog of *S. cerevisiae YKE4,* whose product is a bidirectional zinc transporter located in the endoplasmic reticulum^18^. The number of predicted transmembrane domains in the *C. gattii* Zip proteins ranges from 5 to 9 (Figure S1A). We predicted that these proteins would be localized to the membranes of different subcellular compartments. In agreement with this prediction, analysis using the WoLF PSORT^19^ and SherLoc2^20^ servers predicted that all *C. gattii* Zip proteins were located at the plasma membrane. Phylogenetic analysis employing characterized fungal zinc transporters from the ZIP family as well as the *A. thaliana* Irt1 iron transporter revealed that *C. gattii* Zip1 and Zip2 clustered with the high and low affinity zinc transporters *S. cerevisiae* Zrt1 and Zrt2, respectively (Figure S1B). *C. gattii* Zip4 was located in an independent cluster characterized by the presence of *S. cerevisiae* Yke4 while *C. gattii* Zip3 was not phylogenetically related to the ZIP transporters here analysed (Figure S1B).

**Figure 1:**
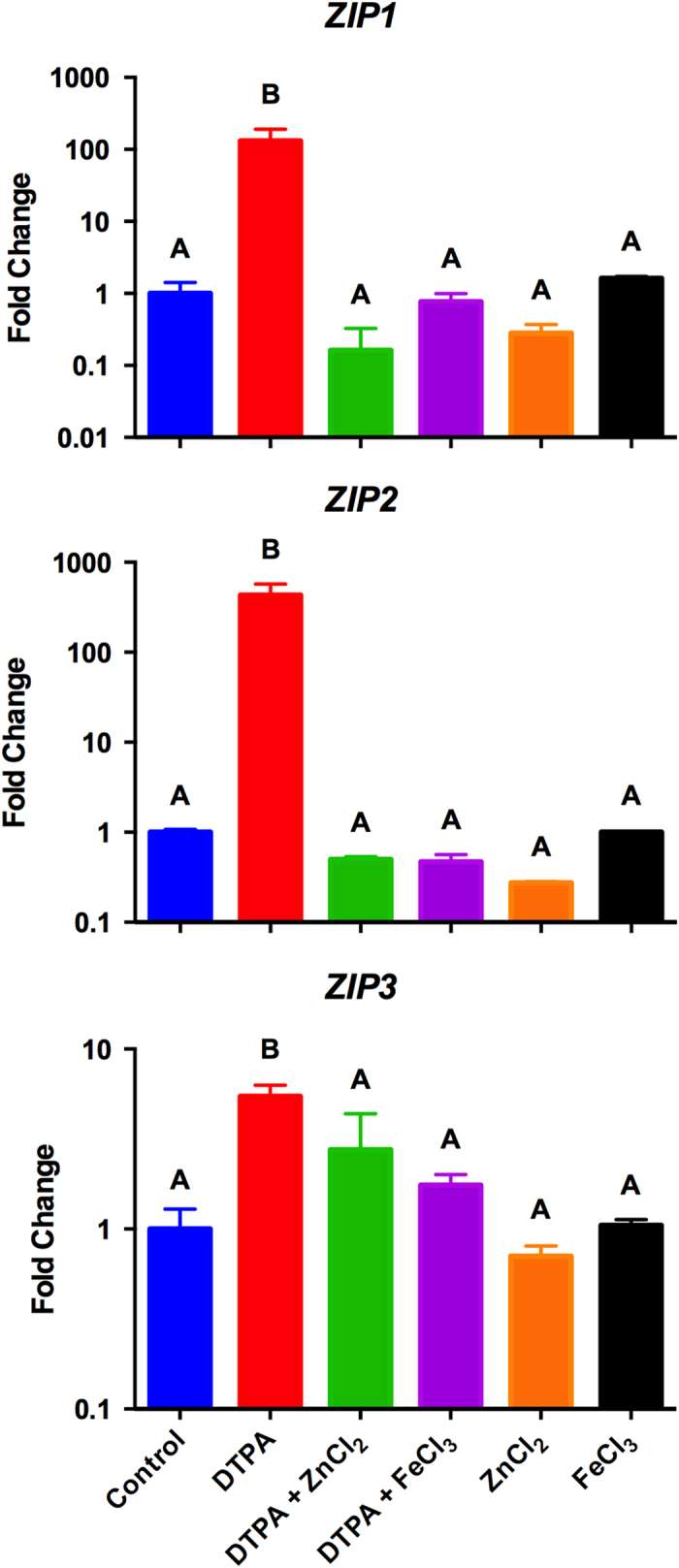
ZIP transcript levels are regulated by metal levels. Quantitative real time RT-PCR of *ZIP* gene transcripts after growth of *C. gattii* in YNB, YNB supplemented with DTPA (100 μM), DTPA (100 μM) added of ZnCl_2_ (400 μM), DTPA (100 μM) added of FeCl_3_ (400 μM), ZnCl_2_ (400 μM) or and FeCl_3_ (400 μM). The measured quantity of the mRNA in each sample was normalized using the *Ct* values obtained for the *ACT1* gene. Data is shown as the mean ± SD from three experimental replicates of three biological replicates. Means with the same letter are not significantly different, as analyzed by one-way ANOVA followed by Tukey multicomparison test.

Transcriptional profiling of the *C. gattii ZIP1*, *ZIP2* and *ZIP3* genes was conducted to evaluate the effects of extracellular zinc levels on *ZIP* family gene expression. Fungal cells were cultured under control conditions in the presence of an extracellular metal chelating agent (diethylene triamine pentaacetic acid - DTPA), under the Zn condition (DTPA supplemented with ZnCl_2_ or ZnCl_2_ without DTPA), and under the Fe condition (DTPA supplemented with FeCl_3_ or FeCl_3_ without DTPA). A significant increase in the transcript levels of all *ZIP* genes was detected when cells were cultured in the presence of DTPA (Fig. 1). In contrast, when zinc or iron was added to the culture medium, the transcript levels of all genes were not statistically distinct from those observed in the control condition (Fig. 1). Therefore, zinc and iron availability regulate the expression of the *ZIP1, ZIP2* and *ZIP3* genes.

### *C. gattii* Zip1 is important for growth under zinc-limiting conditions

**Figure 2:**
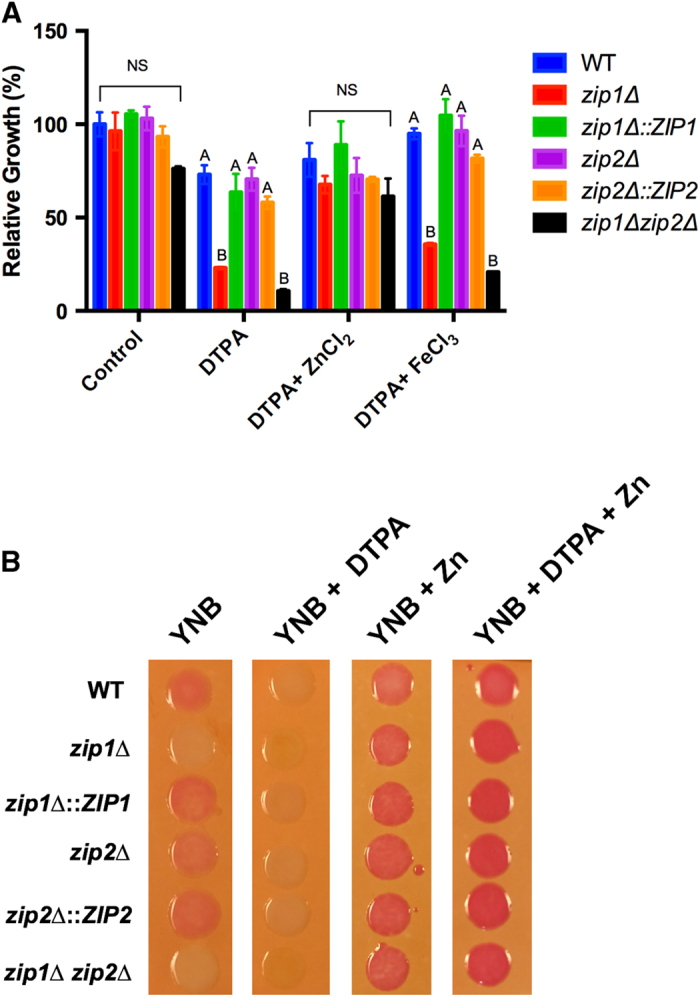
The *C. gattii zip1*Δ and *zip1*Δ *zip2*Δ double mutants are deficient in zinc uptake. A Growth measurement of the WT, *zip1*Δ, *zip1*Δ::ZIP1, *zip2*Δ, *zip2*Δ::ZIP2 and *zip1*Δ *zip2*Δ strains after 48 h of incubation in YNB and YNB supplemented with 100 μM DTPA with and without 100 μM ZnCl_2_ (left panel) or YNB supplemented with 100 μM DTPA with and without 100 μM FeCl_3_ (right panel). Data is shown as the mean ± SD from three biological replicates. For each group, means with the same letter are not significantly different, as analyzed by one-way ANOVA followed by Tukey multicomparison test. **B** Dithizone spot plate assay of *C. gattii* strains grown on YNB with or without 400 μM ZnCl_2_ or YNB supplemented with 100 μM DTPA with or without 400 μM ZnCl_2_.

Functional analyses were conducted for the *ZIP1* and *ZIP2* genes because these genes are targets of the Zap1 transcriptional factor^9^ and are highly regulated by zinc levels. To evaluate the function of Zip1 and Zip2 in *C. gattii*, we constructed null mutants, complemented strains, and a double *zip1*Δ *zip2*Δ mutant strain. Knockout and complementation were confirmed by both Southern blotting and RT-PCR analysis (Figure S2). To evaluate the role of *C. gattii* Zip1 and Zip2 in zinc homeostasis, the sensitivity of the constructed strains to zinc-limiting conditions was evaluated (YNB containing DTPA). Metal deprivation resulted in decreased growth of the *zip1*Δ null mutant and the *zip1*Δ *zip2*Δ double mutant strains (Fig. 2A). This growth reduction in the *zip1*Δ and in the *zip1*Δ *zip2*Δ mutants was recovered when a zinc source was added to the medium containing DTPA (Fig. 2A) or, in the case of *zip1*Δ null mutant, by reintroducing the WT locus into the mutant strain. As DTPA is a broad metal chelator, assays employing iron supplementation were conducted to evaluate if the reversal of *zip1*Δ and the *zip1*Δ *zip2*Δ double mutants growth defect was limited to zinc. Only a partial growth recovery of the *zip1*Δ and of the *zip1*Δ *zip2*Δ mutants was observed when iron was added to DTPA media. Similar results were found for copper and manganese (data not shown). As predicted from the bioinformatic analysis, these results indicate that Zip1 plays an important role in zinc uptake.

To further confirm the hypothesis that Zip1 participates in zinc uptake, we evaluated zinc concentrations in cryptococcal cells. The utilization of the intracellular zinc indicator dithizone generates an intense reddish stain in colonies in the presence of zinc when appropriate intracellular zinc levels are obtained^21^. All strains were incubated on YNB agar plates in the presence of DTPA, DTPA plus zinc or zinc alone. After 18 h of incubation, colonies were transferred to agar plates containing dithizone. The colonies from all strains grown in YNB stained red, with exception of those from the *zip1*Δ mutant and the *zip1*Δ *zip2*Δ double mutant (Fig. 2B). As expected, colonies from all strains grown in the presence of DTPA did not stain with dithizone, and growth in the presence of zinc (with or without DTPA) led to dithizone red staining (Fig. 2B). Requirements for growth during zinc deprivation were also assessed at alkaline pH because the phylogenetic analysis revealed that *C. gattii* Zip2 clustered with *A. fumigatus* ZrfC, which is required for zinc uptake at alkaline pH^22^. As a control, the same analysis was evaluated at pH 5. All of the strains analyzed grew equally well in YNB media, YNB buffered to pH 5 and YNB buffered to pH 8. However, in the presence of the zinc chelator DTPA, only the *zip1*Δ mutant displayed reduced growth (Figure S3). Altogether, these results confirm that Zip1 plays an important role in cryptococcal zinc uptake.

### Compensatory effects on zinc metabolism in the functional mutant ZIP2

**Figure 3:**
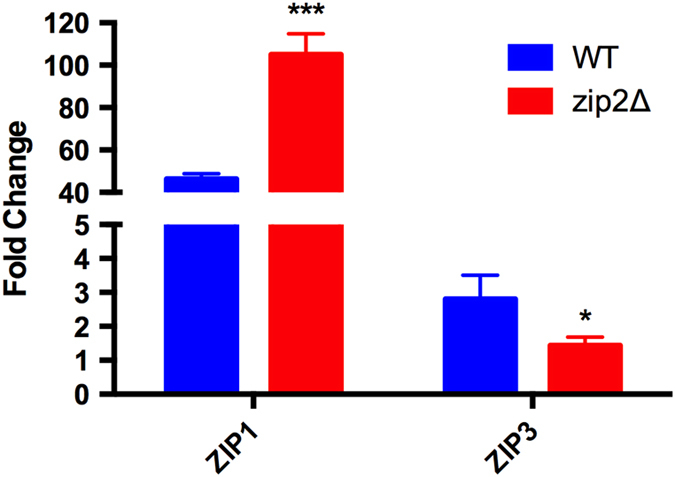
*ZIP1* is overexpressed when *ZIP2* is not functional. Quantitative real-time RT-PCR of *ZIP1* and *ZIP3* gene transcripts after growth of WT or *zip2*Δ mutant cells under control (YNB) and low metal conditions (100 μM DTPA). The measured quantity of the mRNA in each sample was normalized using the Ct values obtained for the *ACT1* gene. Data are shown as the mean ± SD from three experimental replicates of three biological replicates. The asterisks denote statistically significant differences compared to the WT levels (^*^ *P* < 0.05. ^**^ *P* < 0.01^. ***^ *P* < 0.001).

Despite a possible role for *ZIP2* in zinc uptake due to its predicted plasma membrane location, the *zip2*Δ mutant displayed normal growth under zinc-limiting conditions, and its intracellular zinc levels were comparable to the WT strain (Figs. 2A,B). Although *ZIP2* showed the highest expression fold change among the *ZIP* genes in the presence of DTPA (Fig. 1C), its transcript levels under zinc-deprivation conditions were lower than those of *ZIP1* (FPKM values of 10,930.9 for *ZIP1* and 737.7 for *ZIP2* as assessed from transcriptome analysis of the WT strain grown for 2 h in YNB supplemented with the zinc chelator TPEN^9^). These data suggest that Zip1 is the more abundant canonical zinc transporter in *C. gattii* under such conditions. To evaluate whether the lack of *ZIP2* caused a compensatory effect resulting in the upregulation of *ZIP1*, qRT-PCR analysis was performed to compare the transcript levels of the *ZIP1* gene in WT and *zip2*Δ mutant strains grown in control (YNB) or in metal limiting conditions (YNB + DTPA). For both strains, an increase in transcript levels of the *ZIP1* gene was observed in the presence of DTPA, but the relative increase was higher for the *zip2*Δ mutant (nearly 100-fold) when compared to the fold change in WT cells (nearly 50-fold - Fig. 3A). In contrast, an increase in the transcript levels of the *ZIP3* gene was only detected in WT cells (Fig. 3B). These data imply a compensatory effect due to the overexpression of *ZIP1* in the absence of *ZIP2.*

### An imbalance in zinc homeostasis causes an increase in levels of reactive oxygen species

**Figure 4:**
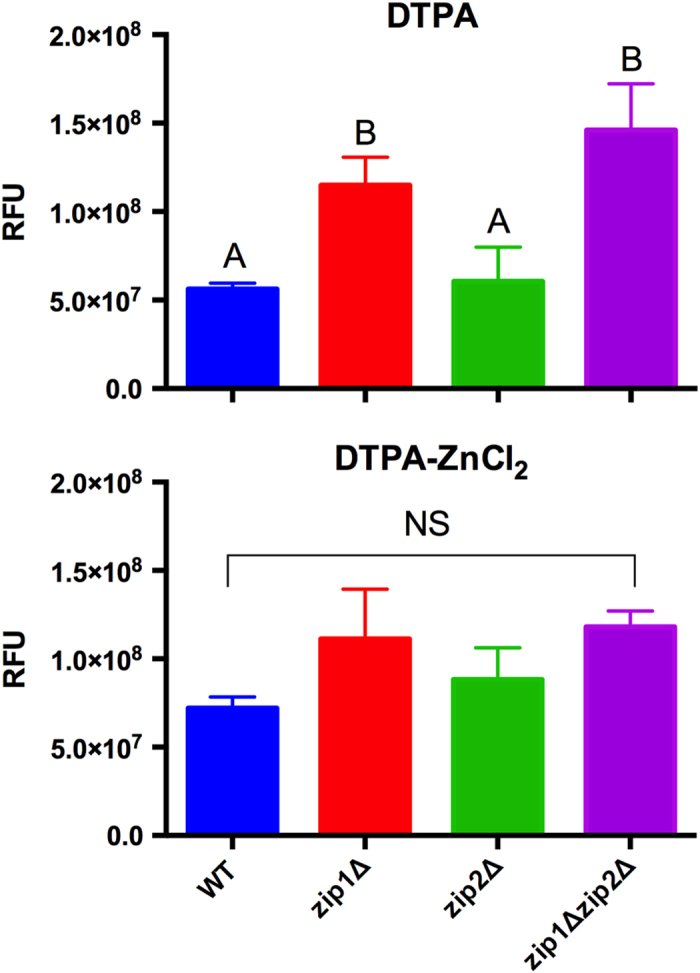
Lack of both *ZIP1* and *ZIP2* cause an imbalance in ROS metabolism. Determination of intracellular ROS levels using the probe CM-H2DCFDA. ROS levels were determined based on fluorometry analyses of green fluorescence. WT, *zip1*Δ mutant, *zip2*Δ mutant and *zip1*Δ *zip2*Δ double mutant strains were cultured for 2 h in YNB with DTPA (100 μM) added or not of ZnCl_2_ (400 μM). The bars represent the cell count normalized fluorescence intensity of three independent biological replicates. Data is shown as the mean ± SD from three biological replicates. Means with the same letter are not significantly different, as analyzed by one-way ANOVA followed by Tukey multicomparison test.

We hypothesized that the *zip1*Δ and *zip1*Δ *zip2*Δ mutant strains could induce the accumulation of intracellular ROS levels because such cells displayed reduced intracellular zinc levels. To test this hypothesis, the WT, *zip1*Δ mutant, *zip2*Δ mutant, and *zip1*Δ *zip2*Δ double mutant strains were incubated for 2 h in medium containing DTPA and DTPA added of ZnCl_2_ and treated with the intracellular ROS probe H2DCFDA, followed by fluorometric analysis. We did not detect differences in ROS levels in cells lacking the *ZIP2* gene compared to WT from DTPA cultures. However, an important increase in ROS levels was detected in the *zip1*Δ and in *zip1*Δ *zip2*Δ mutant strains (Fig. 4). This increase of ROS in cells lacking *ZIP1* or in cells lacking both *ZIP1* and *ZIP2* is somewhat relived when zinc was added to the culture (Fig. 4). These results suggest that zinc uptake by at least Zip1 is necessary to provide zinc to cells to maintain proper ROS homeostasis, however the function of Zip2 in zinc uptake could also be considered.

It is noteworthy that none of the strains analyzed herein showed hypersensitivity to the ROS generating agent hydrogen peroxide or to the glutathione-depleting compound diethyl malate (data not shown). Zinc deficiency generates an increase in oxidative stress in *S. cerevisiae* cells; in these cells, the adaptive response necessary to overcome the damage caused by ROS is mediated by the Zap1p transcription regulator^23^. Our experimental data in *C. gattii* also showed that the acquisition of zinc from the extracellular milieu was important for ROS homeostasis.

### Zip1 and Zip2 are necessary for cryptococcal virulence

**Figure 5:**
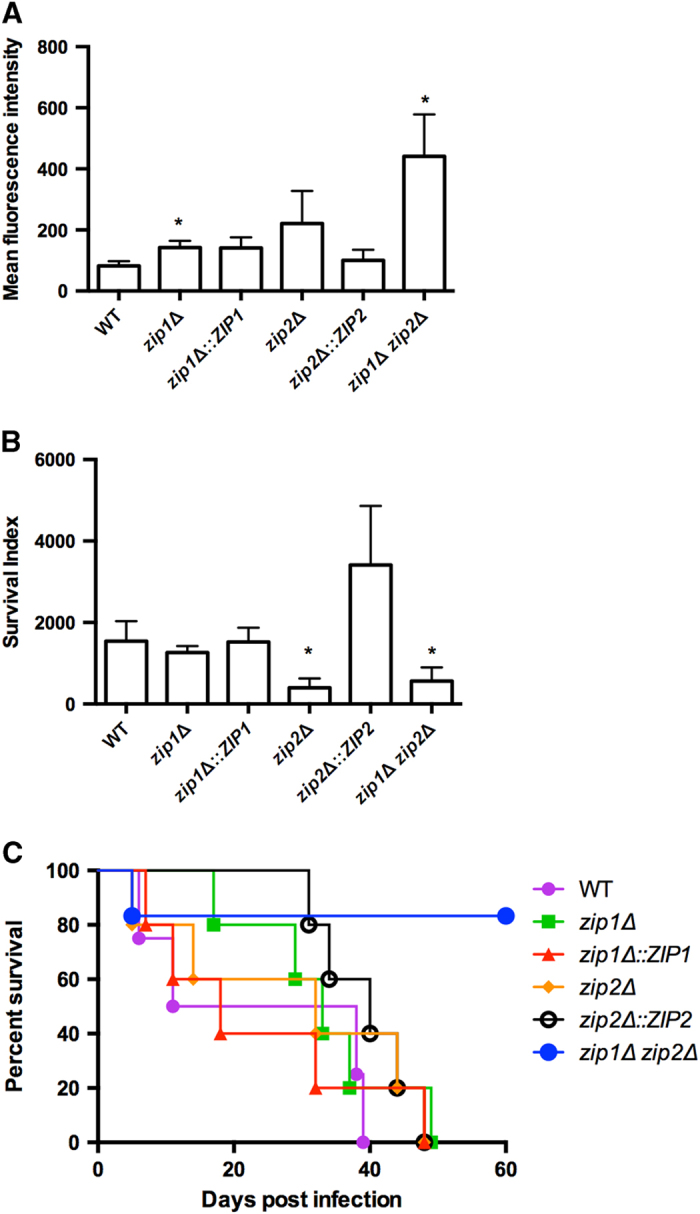
Inactivation of zinc uptake impairs cryptococcal virulence. A Flow cytometry analysis of J774.**A**1 macrophage infection after 2 h of interaction with FITC-labeled *C. gattii* cells. **B** Survival index estimated using the ratio between the CFU following 24 h of interaction and the fluorescence units obtained by flow cytometry following 2 h of interaction. **C** A virulence assay of WT, *zip* mutants, complemented strains and the *zip1*Δ *zip2*Δ double mutant strain in a murine intranasal inhalation infection model.

To evaluate whether zinc transport plays a role in *C. gattii* virulence, we evaluated the levels of the most studied cryptococcal virulence factors in all strains^24^. However, we did not detect any differences in fungal growth at 37 °C, melanization or capsule formation (data not shown). The first step in the pathology of *Cryptococcus* is the interaction between the yeast cells and macrophages^25^. Therefore, it is important to evaluate whether cryptococcal strains lacking the zinc transporter react differently with macrophages. Interaction assays using the generated mutants were performed employing the J774.1 cell line. *C. gattii* cells were labeled with fluorescein isothiocyanate (FITC), and the phagocytosis index was assessed by flow cytometry analysis after 2 h of interaction. We employed an assay in which the index of fluorescence for each macrophage detected with flow cytometry was proportional to the uptake of yeast cells. A higher number of the *zip1*Δ and *zip1*Δ *zip2*Δ mutant strains were internalized by macrophages compared to the WT strain (Fig. 5A). In addition, the capability of *C. gattii* to survive and replicate inside macrophages was affected in the *zip2*Δ and *zip1*Δ *zip2*Δ mutant strains (Fig. 5B) based on the recovery of a lower number of CFUs from the infected macrophages. It is noteworthy that uptake of the *zip2*Δ mutant strain from macrophages was statistically comparable to WT levels, despite a tendency for higher fungal loads in macrophages (Fig. 5A). Considering that the *zip2*Δ mutant exhibited hypersensitivity to macrophages, we propose that Zip2 plays an important role in cryptococcal survival inside macrophages. To further validate the role of the Zip proteins in cryptococcal virulence, we used an intranasal murine model of cryptococcosis. We found enhanced survival only in mice infected with the *zip1*Δ *zip2*Δ strain compared to mice infected with the WT strain (Fig. 5C), despite the hypersensitivity of *C. gattii* strains lacking *ZIP2* to macrophages. Together, these results confirm the importance of proper zinc uptake to cryptococcal virulence.

## Discussion

Transition metals are required for all living organisms. However, the catalytic activity mediated by transition metals can produce a toxic environment, and the levels of these micronutrients must be tightly controlled^26^,^27^. Among transition metals, zinc is used as a cofactor by a large number of proteins^8^,^28^. The complex regulatory mechanism used to acquire and distribute zinc inside fungal cells has been characterized^23^. This system relies on the activity of a group of proteins responsible for zinc transport and redistribution inside the cells, being the best characterized the ZIP and the ZnT family of metal ion transporters^29^. Here, we describe four genes in the *C. gattii* genome that encode the ZIP family of zinc transporters (*ZIP1*, *ZIP2*, *ZIP3* and *ZIP4*), two of which we functionally characterized. The number of zinc transporters from the ZIP family in fungal species ranges from 4 in *S. cerevisiae*^29^ to 8 in *A. fumigatus*^30^. These proteins show different predicted localization and biochemical properties, as exemplified by the high and low specificity of the Zrt1p and Zrt2p proteins from *S. cerevisiae*, respectively^31^,^32^. Here, we further expanded the knowledge of the role of the *ZIP* genes in *Cryptococcus*. Four lines of evidence presented here support that Zip1 is the main zinc transporter in *C. gattii*: (i) its close phylogenetic relationship with *S. cerevisiae* Zrt1p; (ii) the corresponding *ZIP1* transcript levels are highly modulated by zinc availability; (iii) *C. gattii* null mutants of the *ZIP1* gene are hypersensitive to zinc deprivation; and (iv) the deletion of *ZIP1* led to reduced intracellular zinc levels. These same phenotypes and characteristics were not associated with cryptococcal protein Zip2, suggesting that Zip1 is the main ZIP family member responsible for zinc acquisition in *C*. *gattii* cells. *S. cerevisiae* Zrt1p is a high affinity zinc uptake system; *zrt1* mutation eliminated the high affinity activity and led to poor growth under zinc-limiting conditions^31^. Additionally, *S. cerevisiae* Zrt2p is a non-essential gene required for the low affinity zinc uptake system. Notably, the *zrt1 zrt2* double mutant strain is viable^33^. Although *C. gattii* Zip1 was found to be fundamental for zinc uptake, Zip2 appears to play a marginal role. Phylogenetic analysis revealed that *C. gattii* Zip2 clustered with *A. fumigatus* ZrfC, a ZIP family transporter involved in zinc uptake in alkaline environments^22^. Zip2 in both fungi was predicted to be localized to the plasma membrane^22^,^31^. Even in the absence of *ZIP2,* cryptococcal cells were able to grow under zinc deprivation. This may be a consequence of a compensatory effect due to the upregulation of Zip1 in the *zip2*Δ mutant. In accordance, we previously showed that both *ZIP1* and *ZIP2* are under the regulation of the transcription factor Zap1^9^. Nevertheless, this compensatory mechanism is not conserved because the lack of the low affinity transporter Zrt2 did not influence the expression of *ZRT1* in *S. cerevisiae*^32^. These results suggest that *C. gattii* has evolved redundant mechanisms to obtain zinc from the extracellular space.

Cryptococcal virulence is dependent on the proper regulation of zinc homeostasis. Previously, we showed that inactivation of the transcription factor *ZAP1* led to reduced virulence in murine models of cryptococcosis. Null mutants of the *ZAP1* gene expressed levels of melanin and capsule comparable to the WT strain, suggesting that disturbed zinc homeostasis does not interfere with the most common virulence factors. Therefore, the lack of proper zinc uptake is the main cause of the reduced virulence of the *C. gattii zap1* null mutants^9^. The abrogation of zinc uptake led to a drastic reduction in virulence in *C. gattii*, nearly abolishing the mortality rates of mice infected with strains bearing simultaneously inactivated *ZIP1* and *ZIP2* genes. No differences were found in the virulence of strains containing inactivated individual *ZIP1* or *ZIP2* genes, suggesting that *C. gattii* zinc acquisition in the infection milieu relies on the possible functional redundancy of Zip1 and Zip2. The *C. albicans* ortholog of *C. gattii* Zip2 (Zrt1p) is involved in virulence, and its null mutants present reduced growth and development in endothelial cells in a zinc-dependent manner^34^. Similar to *S. cerevisiae*, *C. albicans* also possesses four ZIP protein-coding genes. This demonstrates that some fungal ZIP proteins are individually associated with virulence, but this is not the case for *C. gattii.* A reasonable assumption can be made that *C. albicans* encodes a specialized zinc acquisition system consisting of the secretion of a zinc chelating protein (zincophore) that delivers zinc into Zrt1p for transport^34^. Bioinformatic analysis based on the *C. gattii* genome did not identify an ortholog of the *C. albicans* zincophore coding gene (data not shown).

Zinc has the ability to preclude the redox activity of transition metals, such as copper and iron^35^, and an increase in ROS levels has been observed in zinc-deficient cultured mammalian^12^ and *S. cerevisiae* cells^36^. Herein, we demonstrated that the *zip1*Δ and *zip1*Δ *zip2*Δ mutant strains had low intracellular zinc contents. In accordance, the *zip1*Δ mutant and the *zip1*Δ *zip2*Δ double mutant strains displayed higher intracellular ROS levels. One of the major strategies used by phagocytes to inactivate microbes is the production of toxic ROS^37^. For example, to cope with pathogen invasion, macrophages activate an antifungal response that culminates with the sequestration of exchangeable zinc away from the intracellular pathogens, thereby increasing ROS production^38^. Indeed, the *C. gattii zip1*Δ *zip2*Δ mutant strain was phagocytosed with greater efficiency by macrophages in culture. It is noteworthy that Zip2 alone is important for cryptococcal survival inside macrophages. As iron levels could regulate the expression of this transporter, it is reasonable to infer that it may also participate in iron transport. It is well documented that iron acquisition is a fundamental virulence determinant for *C. neoformans*^39^ and that iron depletion is used as an antifungal activity by macrophages^40^. Therefore, we postulate that the reduced survival of *C. gattii* lacking *ZIP2* could be associated with defects in the acquisition of other metals. However, additional biochemical characterizations of Zip2 are necessary to validate this hypothesis.

In conclusion, this report described the identification and characterization of members of the ZIP family of zinc transporters in *C. gattii*. Two key events were responsible for the observed reduced virulence of the *C. gattii zip1*Δ *zip2*Δ double null mutant in the intranasal murine model of infection: the reduced zinc load in cells and the corresponding increase in intracellular ROS.

### Experimental procedures

#### Ethics statement

The animals were cared for according to the Brazilian National Council for Animal Experimentation Control (CONSEA) and Brazilian College of Animal Experimentation (COBEA) guidelines. Mice were housed in groups of six in filtered top ventilated cages, maintained in a 12 h dark/light cycle and provided with food and water ad libitum. All efforts to minimize animal suffering were made. Before mortality analysis, mice were intraperitoneally anesthetized with 100 mg/kg ketamine and 16 mg/kg xylazine. Mice were analyzed twice daily for any signs of suffering, defined by weight loss, weakness or the inability to eat or drink. Mice were sacrificed following the first signs of suffering. The Universidade Federal do Rio Grande do Sul Ethics Committee for Use of Animals (CEUA - protocol number 19801) approved the use of animals in the present work.

#### Strains and culture conditions

Fungal strains were routinely cultured in YPD media (2% glucose, 2% peptone and 1% yeast extract) incubated at 37 °C in a constant rotation platform. Agar was added at a final concentration of 1.5% when solid media was used, and hygromycin or G418 was added at a final concentration of 100 μg/ml or 200 μg/ml, respectively, for the selection of transformants. The *C. gattii zip1*Δ and *zip2Δ* mutant strains were selected using hygromycin, and the *zip1*Δ::ZIP1 and *zip2*Δ::ZIP2 complemented strains and the *zip1*Δ *zip2*Δ double mutant strain were selected using G418. The medium used for phenotypic assays was yeast nitrogen base (YNB) without amino acids; asparagine was added at a final concentration of 40 mM. J774.A1 macrophage lineage cells were cultured in Dulbecco’s modified Eagle’s medium (DMEM) with 10% heat-inactivated fetal bovine serum (FBS) supplemented with 1 mM l-glutamine, 1 mM sodium pyruvate, and 1% nonessential amino acids (SIGMA) and incubated at 37 °C with 5% CO_2_.

#### Bioinformatic analysis

The gene sequences of the four identified ZIP zinc transporters were retrieved from the Broad Institute *C. gattii* R265 genome database^17^. Prediction of transmembrane helices was conducted by employing the TMHMM tool^41^. Phylogenetic analyses were conducted with the ClustalW alignment in Mega 6^42^ by applying the neighbor-joining method; the tree architecture was inferred from 1,000 bootstraps.

#### Gene knockout and complementation

The sources of the hygromycin and G418 resistance cassettes were plasmids pJAF15^43^ and pJAF1^43^, respectively. The 5’ and 3’ flanking regions of *ZIP1* and *ZIP2* were PCR amplified and gel purified using the Illustra GFX PCR DNA and Gel Band Purification kit (GE Healthcare) and the hygromycin resistance cassette. Each fragment was mixed with SmaI-cleaved pUC18 using the In-Fusion® HD EcoDry™ cloning kit following the manufacturer’s protocol. The reactions were transformed in *Escherichia coli* TG2 cells for blue/white screening selection. For double mutant strain construction, the same protocol was employed with the 5’ and 3’ flanking regions of *ZIP2* and the G418 selection marker cloned into the SmaI site of pUC18 after confirmation that the vector was transformed into the *zip1*Δ mutant strain. For complementation, amplicons encompassing WT *ZIP1* or *ZIP2* flanked by 1 kb on each side were cloned into the EcoRV site of pJAF1. The resulting plasmids were used for transformation of the *zip1*Δ and *zip2*Δ mutant strains. The vectors were transformed into *C. gattii* by biolistic transformation^44^, screening was performed using colony PCR, and the deletion was confirmed by southern blot analysis and semi-quantitative RT-PCR. Primers used in these constructions are listed in Table S1.

#### Zinc deprivation sensitivity test

The zinc sensitivity test was performed following pre-culturing of fungal strains in YPD media overnight at 30 °C. Cells were washed three times with PBS, and the cell density was determined in a Neubauer chamber. A total of 50.000 cells was suspended in 100 μL of YNB (control), YNB supplemented with DTPA (100 μM) in the absence or presence of ZnCl_2_ (100 μM) or FeCl_3_ (100 μM) and distributed into 96 well plates. After 48 h of incubation at 30 °C, OD_600_ was determined in a microplate reader. The relative growth was measured based on WT cell growth in YNB. Statistical analyses were conducted via a two-tailed Student’s t-test or by one-way ANOVA test followed by Tukey multicomparison.

#### Dithizone assay

The intracellular zinc concentration was estimated using the dithizone assay. Dithizone stock solutions and agar plates were prepared as described elsewhere^21^. A sterile nitrocellulose membrane was placed on a YNB agar plate in the presence or absence of DTPA (100 μM) or ZnCl_2_ (400 μM). An aliquot of 5 μl from a standardized cell suspension (OD_600_ = 1) was spot-inoculated onto the nitrocellulose membrane and incubated for 18 h at 30 °C. After incubation, nitrocellulose membranes containing colonies were transferred onto dithizone agar plates, incubated for 1 h in the dark and photographed.

#### Real time RT-PCR analyses

RNA was isolated using Trizol (Invitrogen) after cellular lysis with liquid nitrogen using a mortar and pestle. RNA integrity and concentration were assessed by electrophoresis on a 1% agarose gel and by fluorometry analysis using a Qubit fluorometer and the Quant-iT RNA assay kit according to the manufacturer’s instructions (Invitrogen). cDNAs were prepared from DNase (Promega)-treated total RNA samples (200 ng) with ImProm-II Reverse transcriptase (Promega) using oligo-dT. qRT-PCR was performed on a StepOne Real-Time PCR System (Applied Biosystems) with thermal cycling conditions set with an initial step at 95 °C for 10 min, followed by 40 cycles at 95 °C for 15 s, 55 °C for 15 s and 60 °C for 60 s. Platinum SYBR green qPCR Supermix (Invitrogen) was used as a reaction mix and supplemented with 5 pmol of each primer and 1 μl of the cDNA template for a final volume of 20 μl. All experiments were performed using three independent cultures, and each cDNA sample was analyzed in triplicate for each primer pair. A melting curve analysis was performed at the end of the reaction to confirm the presence of a single PCR product. Data were normalized to actin cDNAs amplified in each set of PCR experiments. Relative expression was determined by the 2^−ΔCt^ method^45^. Statistical analyses were conducted via a two-tailed Student’s t-test or by one-way ANOVA test followed by Tukey multicomparison. The primers used in these analyses are listed in Table S1.

#### ROS measurements

For ROS measurements, cells were cultured overnight in YNB medium at 30 °C in a constant rotation platform. Cells were washed 3 times using phosphate buffer and counted in a Neubauer chamber. An inoculum of 1 × 10^7^ cells/ml was suspended in YNB medium containing DTPA (100 μM) or DPTA added of ZnCl_2_ (400 μM). After incubation for 2 hours at 30 °C in a constant rotation platform, 1 ml of the inoculum was collected, and H2DCFDA (Invitrogen) was added to a final concentration of 10 μM. Cells were incubated in the dark for 2 h, washed with PBS and analyzed by fluorometry using a SpectraMax I3 plate reader fluorometer (Molecular Devices) with the emission and excitation wavelengths set at 488 and 520 nm, respectively. Fluorescence values were normalized to cell count, based on the OD_600_ determination. Statistical analyses were conducted with ANOVA followed by Tukey multicomparison test.

#### Macrophage assays

Phagocytosis assays were conducted to evaluate the susceptibility of the mutant strains to macrophage phagocytosis activity and the resistance of the strains to the antifungal action of phagocytes. One day before the phagocytosis test, an aliquot of 10^6^ J774.A1 cells in DMEM supplemented with 10% FBS was seeded into 12-well culture plates and activated with 100 U/ml INF-γ (Sigma) and 500 ng/mL LPS (Sigma) for 18 h at 37 °C with 5% CO_2_. The *C. gattii* strains were inoculated into YPD and allowed to grow at 30 °C for 18 h. The next day, the *C. gattii* cells were washed 3 times with PBS and opsonized with anti-GXM antibody 18B7 (final concentration of 1 μg/ml) and incubated for 1 h at 37 °C. After opsonization, *Cryptococcus* cells were centrifuged, and the pellet was resuspended in 500 μl of a FITC solution (500 μg/ml) and incubated for 10 min at room temperature. The cells were washed several times with PBS and counted in a Neubauer Chamber. A volume of 1 ml of 1 × 10^7^ cells/ml was added to the wells containing macrophage cells and incubated for 2 h at 37 °C with 5% CO2. After the incubation period, the wells were washed 3 times with PBS and treated with Trypan Blue (Sigma) to reduce FITC fluorescence from non-internalized cryptococci. The cells were collected by scraping and analyzed with a Guava easyCyte Flow Cytometer (Merck Millipore) by measuring the green fluorescence of 5000 events^46^. A second plate under identical conditions was incubated for 24 h to evaluate antifungal activity. After incubation, the wells were washed 3 times with PBS, the cells were lysed with sterile ice-cold water and subsequently plated on YPD plates for CFU determination. The survival index was obtained by normalizing the CFU counts after 24 h of interaction to the fluorescence units obtained in the flow cytometric analysis following 2 h of interaction^47^. All assays were performed in triplicate for each strain. A Student’s t-test was used to determine the statistical significance of the observed differences in fungal survival.

#### Survival assays

The survival assay was performed as previously described^48^. Briefly, the strains were cultured overnight in YPD medium at 30 °C with shaking, then washed three times and re-suspended in PBS. Groups of six female BALB/c mice (4 weeks old) were intraperitoneally anesthetized with 100 mg/kg ketamine and 16 mg/kg xylazine and infected intranasally with 1 × 10^5^ cells in a volume of 50 μl. The mice were monitored twice daily for signals of suffering. The median survival values were calculated using a Kaplan–Meier survival analysis in GraphPad Prism software. Animal studies were approved by the Federal University of Rio Grande do Sul Ethics Committee.

## Additional Information

**How to cite this article**: Schneider, R. O. *et al*. Effects of zinc transporters on *Cryptococcus gattii* virulence. *Sci. Rep.* *5*, 10104; doi: 10.1038/srep10104 (2015).

**Accession codes:** Sequences used for tree construction were derived from *S. cerevisiae* (SGD accession codes Zrt1p, Zrt2p, Zrt3p and Yke4), *A. fumigatus* (NCBI accession codes AAT11930.1 – ZrfA; AAT11931.1 – ZrfB; and EDP50333.1 – ZrfC), and *Arabidopsis thaliana* (NCBI accession code NP_567590.3).

## Supplementary Material

Supplementary Information
