# Supplementary material for: Effects of zinc transporters on Cryptococcus gattii virulence
Source: Sci Rep. 2015 May 7;5:10104. doi: 10.1038/srep10104 (PMC4423424; doi:10.1038/srep10104)
Supplement: Supplementary Information [file srep10104-s1.pdf]

Effects of zinc transporters on *Cryptococcus gattii* virulence

Rafael de Oliveira Schneider[1], Camila Diehl[1], Francine Melise dos Santos[1], Alícia Corbellini Piffer[1], Ane Acosta Wichine Garcia[1], Marcos Iuri Ross Kulmann[1], Augusto Schrank[1,2], Lívia Kmetzsch[1,2], Marilene Henning Vainstein[1,2], Charley C. Staats[1,2,*].

SUPPLEMENTARY MATERIAL

Supplementary Figure 1

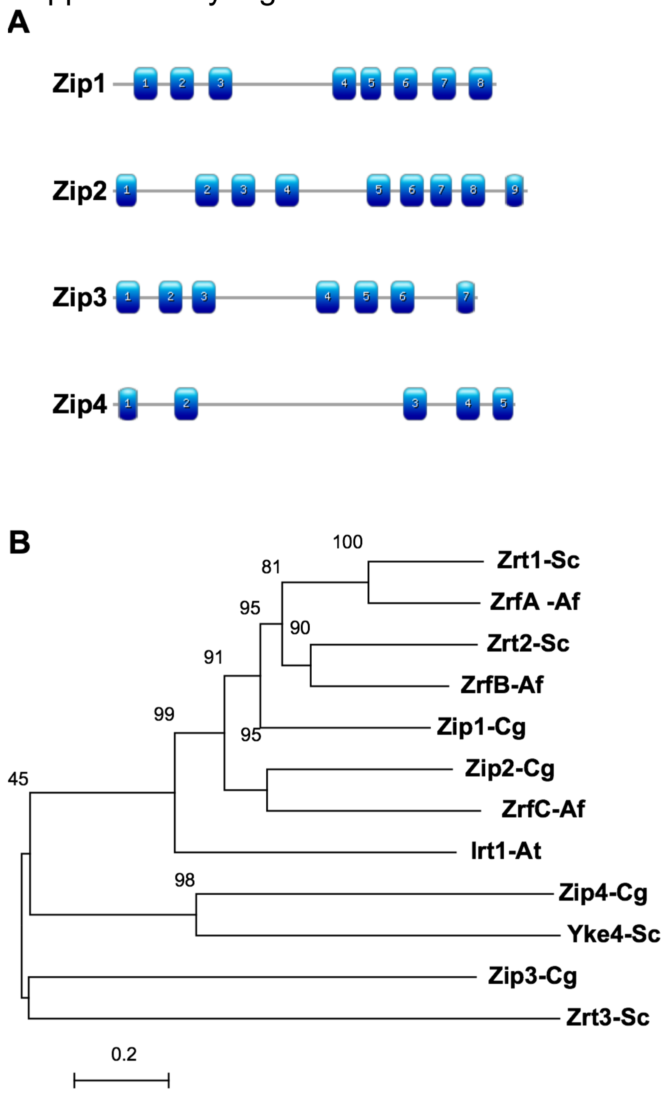

Figure S1. Some characteristics of *C. gattii* zinc transporters of the ZIP family. A Predicted transmembrane helices of the four Zip proteins encoded by the *C. gattii* genome were evaluated with the TMHMM tool and are illustrated as numbered blue boxes. **B** Phylogenetic analysis applying the neighbor-joining method and including Zip zinc transporters sequences from related fungi (Sc – *S. cerevisiae;* Af – *A. fumigatus*; Cg – *C. gattii*) and Irt1 from *A. thaliana*.

Supplementary Figure 2.

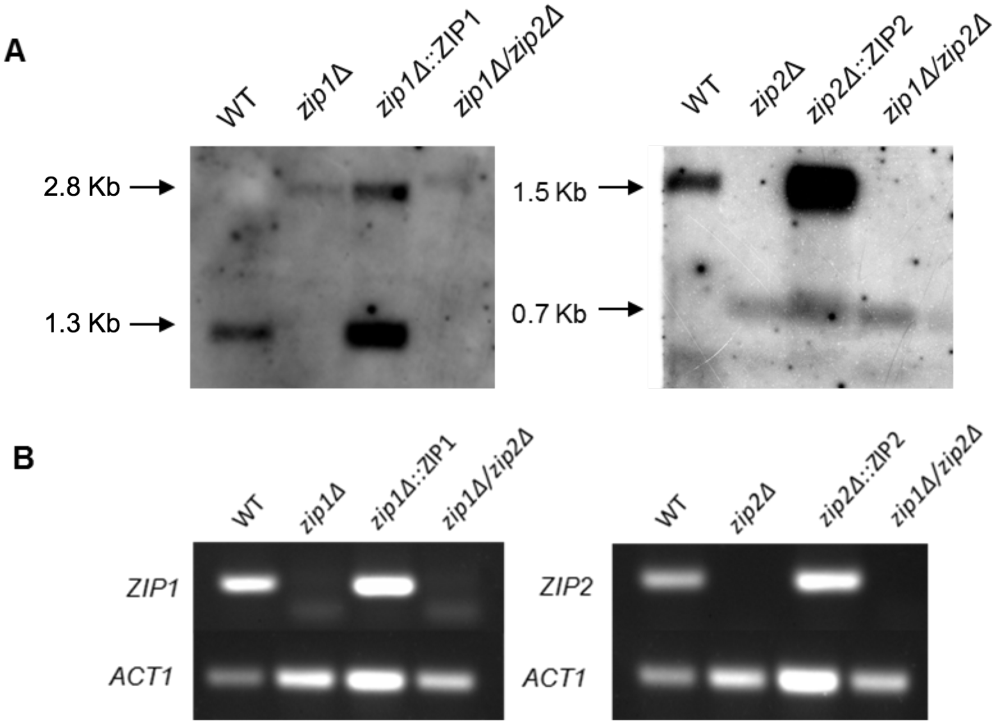

Figure S2. Construction of knockout and complemented strains. A Confirmatory Southern blot. Genomic DNA (10 μg) from the WT, *zip1*Δ mutant, *zip1*Δ::ZIP1 complemented and *zip1*Δ *zip2*Δ double mutant strains were digested with EcoRV. The 5′ gene flanking region was used as a probe for Southern hybridization. DNA (10 μg) from the WT, *zip2*Δ mutant, *zip2*Δ::ZIP2 complemented and *zip1*Δ *zip2*Δ double mutant strains were digested with the PstI restriction enzyme. In this experiment, the 3′ gene flanking region was used as a probe for Southern hybridization. Numbers at the left indicate the hybridization signal sizes based on the position of the molecular size marker. **B** Semi-quantitative RT-PCR using cDNA from the WT, *zip1*Δ mutant, *zip1*Δ::ZIP1 complemented and *zip1*Δ *zip2*Δ double mutant strains (left panel). RNA samples from the WT, *zip2*Δ mutant, *zip2*Δ::ZIP2 complemented and *zip1*Δ *zip2*Δ double mutant strains (right panel) were used

as templates for a reaction employing reverse transcriptase. The upper panel shows the *ZIP* amplicons, and the lower panel shows the *ACT1* amplicons used as loading controls.

Supplementary Figure 3

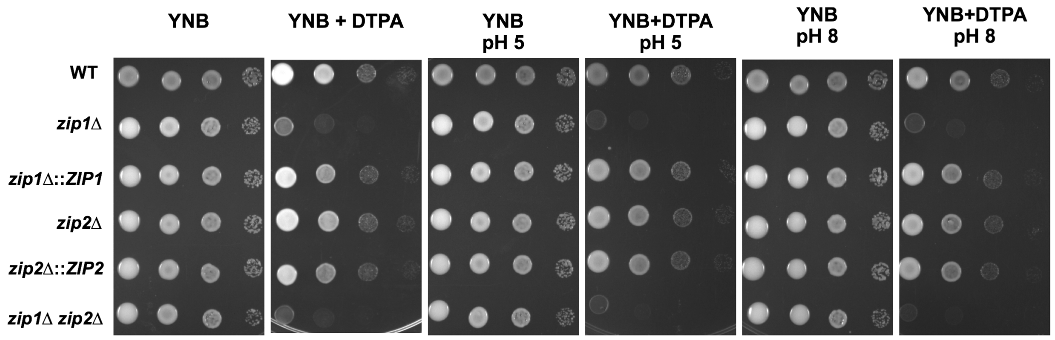

Figure S3. *C. gattii ZIP2* is not required for cryptococcal growth at different pHs Growth of the WT, *zip1*Δ, *zip1*Δ::ZIP1, *zip2*Δ, *zip2*Δ::ZIP2 and *zip1*Δ *zip2*Δ strains in YNB, YNB supplemented with 100 µM DTPA with or without 400 µM $ZnCl_2$ at alkaline pH (8) or a control pH (5) obtained using 20 mM phosphate buffer.

Supplementary Table 1: List of primers used in this work

**Primer name**	**Sequence (5’-3’)**	**Purpose**
**ZIP1-5F**	AAAATAGGGATAACAGGGTAATGGAAAATGTGGATTGGATGG	5’-flanking amplicon of *ZIP1* knockout construct
**ZIP1-5R**	GGGGACAAGTTTGTACAAAAAAGCAGGCTATGAAATGACTGACGGGTCGTT	5’-flanking amplicon of *ZIP1* knockout construct
**ZIP1-3F**	GGGGACCACTTTGTACAAGAAAGCTGGGTACATTGTAGAAAATCCGTGAGAAG	3’-flanking amplicon of *ZIP1* knockout construct
**ZIP1-3R**	AAAAATTACCCTGTTATCCCTACGACAGGCTCTCAACGAGAC	3’-flanking amplicon of *ZIP1* knockout construct
**ZIP2-5F**	AAAATAGGGATAACAGGGTAATAGCAAGATAGGGGCTTACCC	5’-flanking amplicon of *ZIP2* knockout construct
**ZIP2-5R**	GGGGACAAGTTTGTACAAAAAAGCAGGCTATCATGCATCAGCGTGAGGA	5’-flanking amplicon of *ZIP2* knockout construct
**ZIP2-3F**	GGGGACCACTTTGTACAAGAAAGCTGGGTAAAAAAGGGGGAAGAGTTTGG	3’-flanking amplicon of *ZIP2* knockout construct

**ZIP2-3R**	AAAAATTACCCTGTTATCCCTAT GAGTGGGAGCATGGAACT	3'-flanking amplicon of *ZIP2* knockout construct
**ZIP1compF**	AGGGCGAATTCTGCAGATGCAA GAGACTAAGTCAGAGACTAAGT CAGAATCCA	*ZIP1* amplicon for complementation
**ZIP1compR**	CGGCCGCCAGTGTGATGGATCT CTTCGACAGGCTCTCAAC	*ZIP1* amplicon for complementation
**ZIP2compF**	AGGGCGAATTCTGCAGATTTAC CCGCAGAGGCTCTTT	*ZIP2* amplicon for complementation
**ZIP2compR**	CGGCCGCCAGTGTGATGGATCT GGAGGACGACGATGAAGAA	*ZIP2* amplicon for complementation
**CGACTF**	CGGTATCGTCACAAACTGG	Amplification of *ACT1* for RT-PCR/qRT-PCR
**CGACTR**	GGAGCCTCGGTAAGAAGAAC	Amplification of *ACT1* for RT-PCR/qRT-PCR
**CNBG5361F**	GTGTAGCCCTTCTTTTCTCC	Amplification of *ZIP3* for qRT-PCR
**CNBG5361R**	CAATCCTGTCCGTTGGTAC	Amplification of *ZIP3* for qRT-PCR
**CNBG6066F**	GCTGAAGTCGCCGCTTATC	Amplification of *ZIP1* for qRT-

		PCR
**CNBG6066R**	GGGAGGGATGGATGTGATG	Amplification of *ZIP1* for qRT-PCR
**CNBG2209F**	GTCATATTGGGCAAACTGG	Amplification of *ZIP2* for qRT-PCR
**CNBG2209R**	AGGGGCAACAGACTCATAG	Amplification of *ZIP2* for qRT-PCR
